# Qualitative and quantitative comparison of two semi-manual retinal vascular density analyzing methods on optical coherence tomography angiography images of healthy individuals

**DOI:** 10.1038/s41598-023-44234-z

**Published:** 2023-10-09

**Authors:** Orsolya Angeli, Dorottya Hajdu, Aniko Jeney, Balint Czifra, Balazs Vince Nagy, Tamas Balazs, Dora Jakaboczkine Nemoda, Gabor Mark Somfai, Zoltan Z. Nagy, Tunde Peto, Miklos Schneider

**Affiliations:** 1 Department of Ophthalmology, Semmelweis University, Budapest, Hungary; 2 Department of Ophthalmology and Optometry, Vienna Clinical Trial Center (VTC), Medical University of Vienna, Vienna, Austria; 3 Department of Ophthalmology, Flor Ferenc Hospital, Kistarcsa, Hungary; 4 Department of Mechatronics, Optics and Mechanical Engineering, Budapest University of Technology and Economics, Budapest, Hungary; 5 Healthware Ltd, Budapest, Hungary; 6 Department of Ophthalmology, Stadtspital Zurich, Zurich, Switzerland; 7 Spross Research Institute, Zurich, Switzerland; 8 Centre for Public Health, Queen’s University Belfast, Belfast, UK; 9 Research Unit of Ophthalmology, Department of Clinical Research, University of Southern Denmark, Odense, Denmark; 10 Department of Ophthalmology, Rigshospitalet Glostrup, Valdemar Hansens Vej 1-23, 2600 Glostrup, Denmark

**Keywords:** Image processing, Medical research, Retina, Medical imaging

## Abstract

The aim of this study was to evaluate qualitative and quantitative differences in vascular density analysis of an established and a novel alternative for post-processing on optical coherence tomography angiography (OCTA) images in healthy individuals. OCTA examinations of 38 subjects were performed. After extracting the images, two semi-manual post-processing techniques, the already established Mexican hat filtering (MHF) and an alternative, the Shanbhag thresholding (ST) were applied. We assessed Vessel Density (VD), Skeleton Density (SkD) and Vessel Diameter Index (VDI). We analyzed the results in order to establish similarities or potentially relevant differences. Regarding SkD and VD, MHF generally gave higher values than ST. Simultaneously, mean values were also predominantly higher by MHF; however, standard deviations (SD) were higher by ST (range [mean ± SD]: 0.054 ± 0.038 to 0.134 ± 0.01 and 0.134 ± 0.095 to 0.362 ± 0.028 vs 0.012 ± 0.014 to 0.087 ± 0.03 and 0.039 ± 0.047 to 0.4 ± 0.095 for SkD and VD with MHF vs SkD and VD with ST, respectively). Values of VDI were considerably higher with ST than with MHF, while standard deviation was still significantly higher with ST (range [mean ± SD]: 2.459 ± 0.144 to 2.71 ± 0.084 and 2.983 ± 0.929 to 5.19 ± 1.064 for VDI with MHF and ST, respectively). The noise level reduction of the two methods were almost identical (noise levels: 65.8% with MHT and 65.24% with ST). Using MHF, the vascular network gets more fragmented by an average of 40% compared to ST. Both methods allow the segmentation of the vascular network and the examination of vascular density parameters, but they produce largely inconsistent results. To determine if these inconsistent results are clinically meaningful, and which method is more suitable for clinical use, our results provide further evidence that detailed understanding of the image analysis method is essential for reliable decision making for patients with retinal pathology. For longitudinal monitoring, use of the same image processing method is recommended.

## Introduction

Optical coherence tomography angiography (OCTA) is an imaging modality based on spectral-domain or swept-source OCT technology that enables non-invasive, dye-free, three-dimensional analysis of the retinal and choroidal vessels^1^. It combines structural information with data on detectable blood flow in the retina. Since its introduction to clinical routine, OCTA has become a widely used tool in the diagnosis and management of several retinal disorders^1,2^.

In the recent past, several publications have discussed the differences between OCTA devices and presented different vascular density assessment methods^3–10^. Results obtained by different OCTA devices are difficult to compare because both the segmentation algorithms and the analysis software used by the system may be built on different principles.

The purpose of the present study was to evaluate and compare qualitative and quantitative differences in vascular density (VAD) analysis of a previously described semiautomated post-processing method (Mexican hat filtering—MHF) with an alternative new method (Shanbhag thresholding—ST), which is not common in ophthalmological image analysis. In order to minimize the variables in the process, the same original images obtained by the same OCTA device were used.

## Materials and methods

### Subjects

Thirty-eight eyes of 38 healthy volunteers (age range: 24–83 years, 17 males and 21 females) were enrolled in this study. Exclusion criteria were any history or clinical evidence of retinal disease or glaucoma, previous ocular surgery or laser photocoagulation and optical media opacities that might influence image quality.

### Image acquisition and processing

Subjects were scanned using the Zeiss Cirrus HD-OCT 5000 AngioPlex spectral-domain OCT device (Carl Zeiss Meditec Inc., Dublin, CA, USA). The device performs each acquisition at a speed of 68 kHz (68,000 A-scans per second), using an 840-nm superluminescent diode with a bandwidth of 45 nm. For the visualization of the microvasculature, the device uses the optical microangiography (OMAG)^3^ segmentation algorithm, which uses the entire OCT signal (both amplitude and phase aspects). The built-in FastTrac™ eye tracker helps to minimize the effects of involuntary eye movements^8^. The AngioPlex is able to separately visualize vessels of the superficial retina, the deep retinal layer, the choriocapillaris and other large choroidal vessels, similarly to other OCTA systems^1^.

A 6 × 6 mm square area, centered on the fovea was scanned in all study subjects in both eyes. The vascular plexuses were segmented using the built-in software of the device. For further analysis, images of the superficial plexus were used. The images were carefully checked individually for quality by two investigators (O.A., M.S.) to ensure they were well-centered, artefact-free and did not contain any segmentation errors. Disagreements were resolved by discussion. The right eye of each patient was included, and in case of suboptimal image quality, scans of the left eye were used.

Following image acquisition, we used two different post-processing methods, Mexican hat filtering (MHF) and Shanbhag thresholding (ST) on each image and assessed three different parameters, such as Vessel Density (VD), Skeleton Density (SkD) and Vessel Diameter Index (VDI).

### Processing method #1—Mexican hat filtering

The principles of this image processing method are described in detail by Kim et al.^6^ First, we imported the OCTA images from the OCT machine to GIMP (software version 10.4, The GIMP Team, Charlotte, NC, USA), a digital image processing software and converted them to 16-bit tiff files. We used ImageJ (software version 1.52a, ImageJ developers, USA) for additional manipulations.

In the next step we filtered our images. We reduced the noise with a 2 × 2 pixel-sized minimum filter. The transformation replaced the actual pixel's value with that of the darkest one in the running window. Typically, when used with white background, this filter widens thin black lines on white surfaces. As our images had black background with white shapes, it works the opposite way: eliminates the small, unnecessary white noise^6^.

The foveal avascular zone (FAZ) was removed manually. In ImageJ, we used the tool “freehand selection”, and then the “cut” function on the selected area. Since this step is subject to the evaluator’s interpretation, further error analysis was needed (described below).

**Figure 1:**
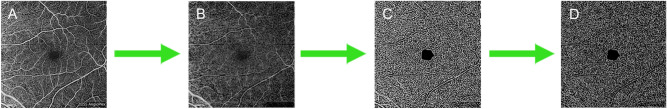
The steps of image processing with Mexican hat filtering method: (**A**) Original image, (**B**) Minimum filtered image with removed avascular zone, (**C**) Mexican hat filtered image, (**D**) Skeletonized image.

The next task was to binarize the sample. For this step, the Mexican hat filtering (MHF) was used (Fig. 1). This filter applies a Laplacian of Gaussian filter to a 2D image, approximated by derivatives of Cardinal B-Splines. This is a built-in filtering function in ImageJ. We used MHF to derive representative expansion data with binarized values. Finally, we used skeletonization on the processed image to gain length-type information^11^. This is also a built-in function in ImageJ^9,10^. Figure 1 shows an example of the applied steps and the resultant images.

### Processing method #2—Shanbhag thresholding

In this image processing method, we used an automatic thresholding technique proposed by Shanbhag^12^. The method was adopted by our research team for the use of OCTA image post-processing and analysis. The Shanbhag thresholding (ST) is also a built-in function of the ImageJ software.

**Figure 2:**
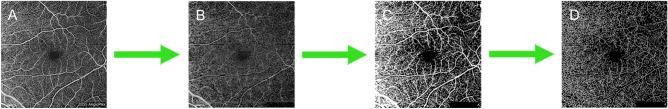
The steps of image processing with Shanbhag thresholding method: (**A**) Original image, (**B**) Minimum filtered image, (**C**) Shanbhag thresholded image with automatically removed avascular zone, (**D**) Skeletonized image.

The first two steps were identical to those in the MHF method (i.e., image import and 16-bit conversion then application of a minimum filter). Subsequently, we performed Shanbhag thresholding (Fig. 2). The Shanbhag method is based on a dynamic thresholding technique. The algorithm determines the incidence rate of grey level of the pixels and calculates a new thresholding level based on the distance from the standard threshold. Manual elimination of the avascular zone is not needed, because this process removes it automatically^12^. In the final step, we skeletonized the image.

### Quantitative analysis

**Figure 3:**
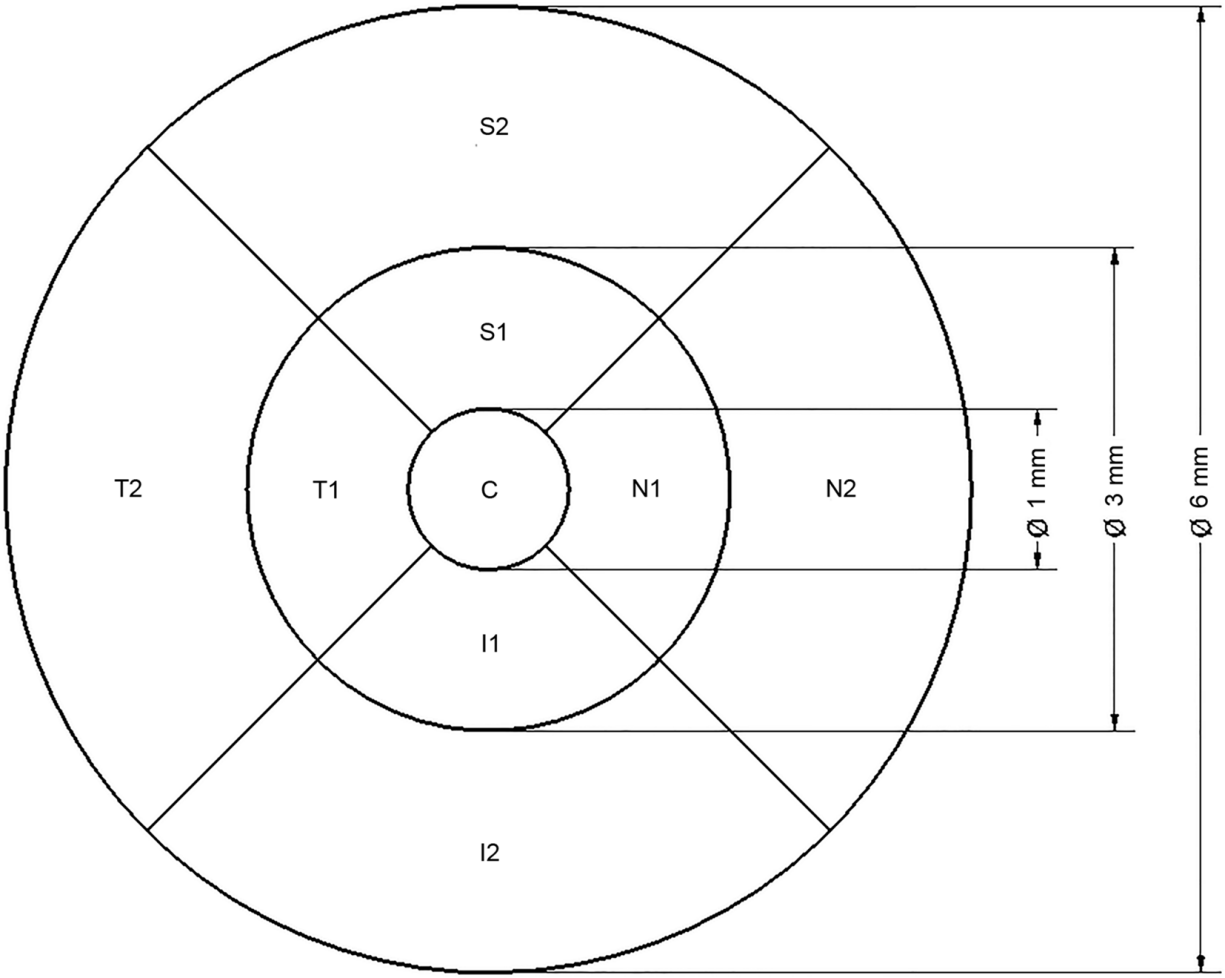
The nine sectors based on the Early Treatment Diabetic Retinopathy Study (ETDRS). Circles are centered around the fovea with diameters of 1, 3 and 6 mm, respectively. Indexes in the sectors refer to their locations as follows: S = superior, N = nasal, I = inferior, T = temporal. Sectors marked with ‘1’ (S1, N1, I1 and T1) are in the inner circle, sectors marked with ‘2’ (S2, N2, I2 and T2) are in the outer circle. Sector ‘C’ represents the central area. This figure illustrates a right eye. The sector numbers in the text correspond to the grid sectors on this illustration as follows: 1 = C, 2 = I1, 3 = T1, 4 = S1, 5 = N1, 6 = I2, 7 = T2, 8 = S2, 9 = N2.

Post-processed images obtained by both methods were quantitatively analyzed. We evaluated Vessel density (VD), Skeleton density (SkD), and Vessel diameter index (VDI) in all nine Early Treatment Diabetic Retinopathy Study (ETDRS)^13^ sectors (Fig. 3).

#### Vessel density (VD)

VD represents the surface of the blood vessels, and it is calculated as a unitless proportion of the total image area occupied by the detected OCTA signal (binarized as white pixels) compared to the total area of retina (total number of pixels) depicted on the binarized image.

$$\mathrm{VD}=\frac{{(\mathrm{white\, pixels})}^{2}}{{(\mathrm{total\, number \,of\, pixels})}^{2}}$$

#### Skeleton density (SkD)

SkD represents the length of blood vessels based on the skeletonized SD-OCTA image, calculated as the following:

$$\mathrm{SkD}=\frac{(\mathrm{white\, skeletonized\, pixels})}{{(\mathrm{total\, number\, of\, pixels})}^{2}}$$

#### Vessel diameter index (VDI)

VDI informs about the actual blood supply of the retina, and it is calculated using the binary blood vessel image and the skeleton image to yield the average vessel volume in the SD-OCTA image (pixels) as follows:

$$\mathrm{VDI}=\frac{{(\mathrm{white\, pixels})}^{2}}{(\mathrm{white\, skeletonized\, pixels})}=\frac{\mathrm{VD}}{\mathrm{SkD}}$$

### Quality assessment and statistical analysis

Following image acquisition, post-processing and data collection, statistical analysis was done.

As the ETDRS grid needed to be placed manually on each image to fit the FAZ correctly, an error calculation was performed. The ETDRS grid was placed four consecutive times on the OCTA images. Error calculation was performed by identifying the white pixels in the four inner (temporal, nasal, superior, and inferior) sectors and comparing the corresponding sectors among the 4 different placements.

As previously mentioned, when using the MHF, we had to make a freehand selection to outline the boundaries of the FAZ. This can also cause errors in the inner sectors. Error calculation was done by selecting the FAZ manually 5 times and counting the average of the differences in white pixel count compared to all the white pixels in the zone. With the ST, this error calculation was not necessary because the technique marked out the FAZ area automatically.

We compared the two methods from the aspect of noise level as well. Measurement of the noise was done by counting the white pixels of the processed images and comparing them to all pixels of the original images.

The number of separated vascular segments is also an important aspect. Without vascular disorders, blood vessels should form one, contiguous segment. Due to the different filters and image-processing methods, different noise artefacts may appear on the image, and therefore, the vascular network can become fragmented into more separated segments. With increasing noise, the quality of the sample gets worse, and the vascular network gets more fragmented. The number of segments was counted in each processed image by MATLAB (version R2017B, The MathWorks, Natick, Massachusetts, USA), after which they were averaged over all processed images for each method individually.

In the case of all measured parameters, the mean and standard deviation values were calculated in every sector, and the distributions were compared by box plots. The degree of agreement of the two processing methods was analyzed with Bland–Altman plots^14^ in all parameters and sectors. In every case, the difference of measurements was calculated by extracting the value of the ST parameter from the value of MHF parameter. In addition to the mean difference, the limits of agreement (± 1.96*SD) were also calculated. The assumption of normality of the difference scores was checked graphically in every case. Statistical significance of the mean difference was analyzed by the 95% confidence interval^15^.

Statistical calculations were carried out with the R system (R Core Team, Vienna, Austria, software version 4.0.4)^16^ using the following packages: data.table^17^, BlandAltmanLeh^18^, ggplot2^19^ and ggpubr^20^.

### Ethics approval and consent to participate

This prospective, observational pilot study was carried out at the Department of Ophthalmology, Semmelweis University, Budapest, Hungary. The study was conducted in accordance with the ethical standards stated in the Declaration of Helsinki. The study protocol was approved by the National Institute of Pharmacy and Nutrition in Hungary (Approval No.: OGYÉI/1253/2017) and registered prospectively at ClinicalTrials.gov (ID: NCT03590899). The patients were fully informed about the examinations and provided written consent.

## Results

Error calculation among the manually placed ETDRS sectors yielded a very low value, average error was 0.58%. Using the MHF method, average error in the FAZ and inner sectors due to the freehand selection was 3.5% and 0.5%, respectively.

From the aspect of noise level reduction, the two methods were almost identical. The noise levels with the MHF and the ST were 65.8% and 65.24%, respectively.

In each processed image, the number of separated segments was counted and averaged. We found that using the MHF, the vascular network gets more fragmented by an average of 40% as compared to the ST method (an average of 3999 ± 469 vs 2390 ± 361 separated segments with MHF and ST, respectively).

Tables 1 and 2 show detailed tabulation of VD, SkD, and VDI parameters calculated by MHF and ST in all ETDRS sectors.

**Table 1:** Parameters calculated by Mexican hat filtering.

	ETDRS Sectors								
	1	2	3	4	5	6	7	8	9
SkD Mean (SD)	0.054 (0.038)	0.119 (0.017)	0.12 (0.014)	0.12 (0.013)	0.118 (0.015)	0.124 (0.012)	0.134 (0.01)	0.123 (0.011)	0.117 (0.011)
VD Mean (SD)	0.134 (0.095)	0.31 (0.045)	0.308 (0.038)	0.313 (0.034)	0.304 (0.043)	0.334 (0.033)	0.362 (0.028)	0.332 (0.028)	0.304 (0.029)
VDI Mean (SD)	2.459 (0.144)	2.603 (0.089)	2.567 (0.064)	2.606 (0.062)	2.568 (0.069)	2.702 (0.092)	2.71 (0.084)	2.693 (0.079)	2.613 (0.064)

*ETDRS* Early treatment of diabetic retinopathy study, *SkD* skeleton density, *VD* vessel density, *VDI* vessel diameter index, *SD* standard deviation.

**Table 2:** Parameters calculated by Shanbhag thresholding.

	ETDRS Sectors								
	1	2	3	4	5	6	7	8	9
SkD Mean (SD)	0.012 (0.014)	0.047 (0.03)	0.044 (0.026)	0.05 (0.026)	0.043 (0.027)	0.063 (0.03)	0.087 (0.03)	0.06 (0.026)	0.041 (0.022)
VD Mean (SD)	0.039 (0.047)	0.214 (0.106)	0.181 (0.09)	0.221 (0.09)	0.175 (0.1)	0.302 (0.109)	0.4 (0.095)	0.289 (0.093)	0.19 (0.078)
VDI Mean (SD)	2.983 (0.929)	4.704 (0.926)	4.465 (0.945)	4.85 (1.096)	4.422 (0.899)	5.18 (1.064)	4.881 (1.093)	5.163 (1.165)	5.064 (1.031)

*ETDRS* Early treatment of diabetic retinopathy study, *SkD* skeleton density, *VD* vessel density, *VDI* vessel diameter index, *SD* standard deviation.

**Figure 4:**
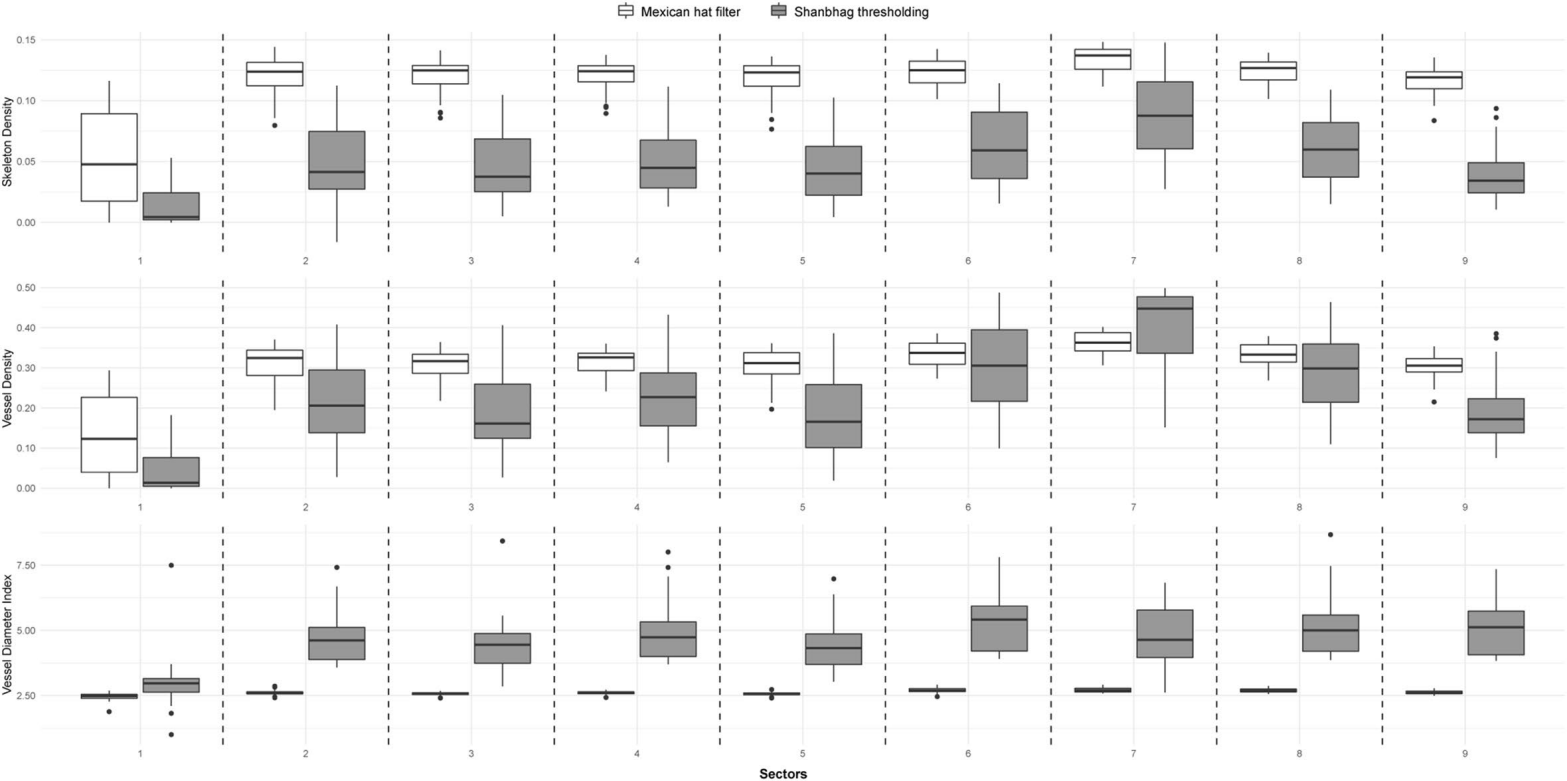
Comparisons of Skeleton Density, Vessel Density, and Vessel Diameter Index parameters with Mexican hat filter and Shanbhag thresholding. Sectors: ETDRS sectors, see Fig. 3. for the explanation of those.

Regarding the SkD and VD parameters, the median, lower and upper quartile values of MHF were generally higher than those of ST (Fig. 4). Exceptions were found only in the case of VD parameter. In sector 6 only the upper quartile while in sector 7 the median and the upper quartile were higher in the case of ST. Simultaneously, mean values were also higher with MHF (excluding VD parameter in sector 7); however, standard deviations were higher with ST.

In contrast to the above, we could observe that the previously mentioned values with VDI were considerably higher with ST than with MHF, while standard deviation was still considerably larger with ST.

The results of sector 1 were significantly different from all other sectors due to the physiologically very low vascular density of the foveal avascular zone.

**Figure 5:**
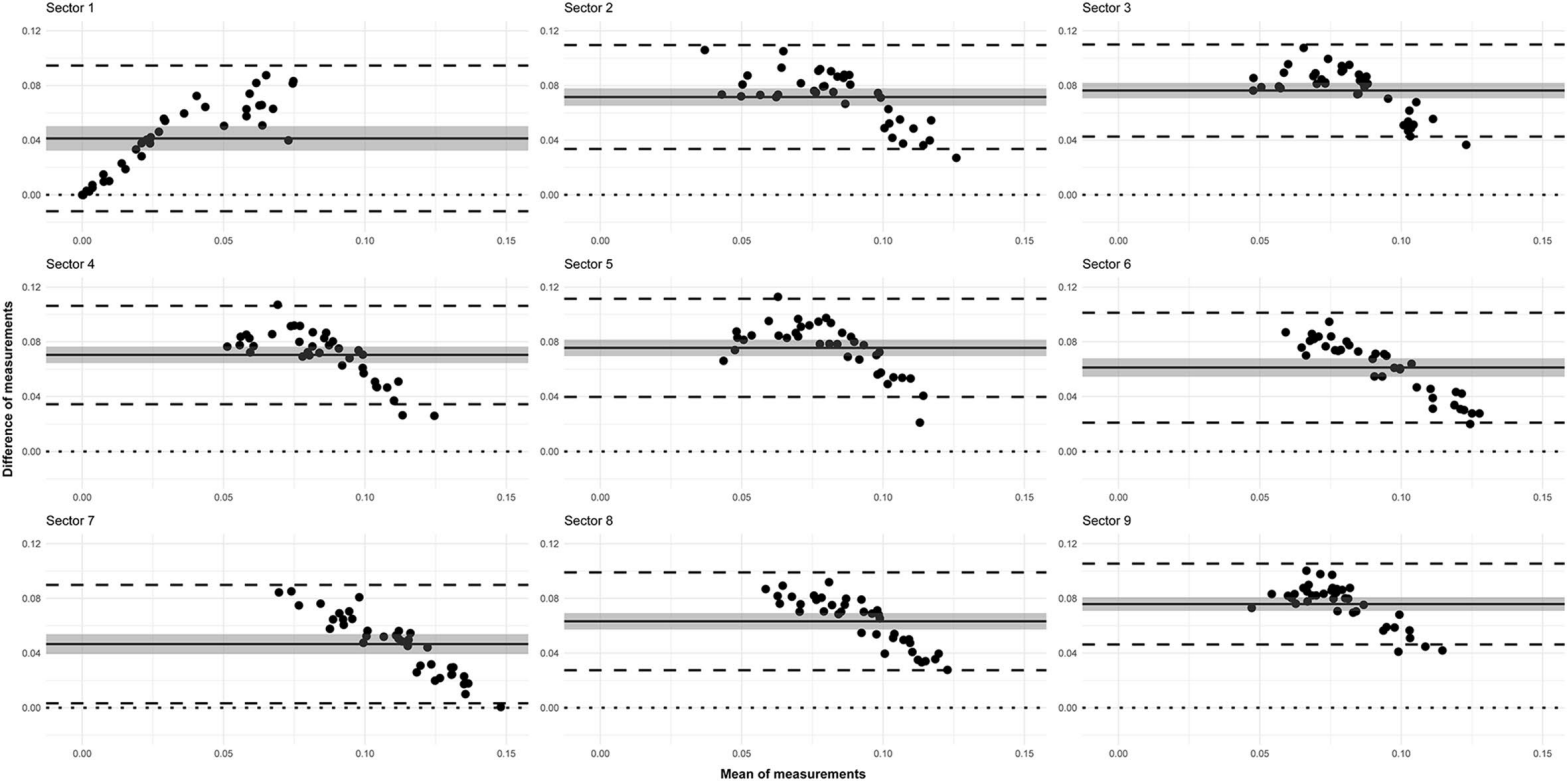
Mexican hat filter and Shanbhag thresholding comparison using the SkD parameter. Bland–Altman plots in the 9 ETDRS sectors (see Fig. 3. for the explanation of the sectors). The x axis represents the mean of measurements, the y axis represents the difference of measurements for each graph, individually. The solid line indicates the mean of the differences; the shade around the solid line indicates the 95% confidence interval of mean difference; the upper and lower dashed lines indicate the upper and lower limits of agreement (LA), whereas the dotted line indicates zero.

In agreement with the previous descriptive results, SkD values obtained after MHT were consistently higher than those after ST on the Bland-Altmann plots as well (Fig. 5).

Based on this observation and the shape of the point cloud, we could conclude that (except for sector 1, for reasons described earlier) there is a negative correlation between the means and differences of the two methods. This means that the higher the mean of the measurements is, the smaller the difference will be. The lower limit of agreement contains zero only in sector 1, and it is located near zero in sector 7. Based on the 95% confidence interval, the mean of differences was significantly different from zero in all sectors.

**Figure 6:**
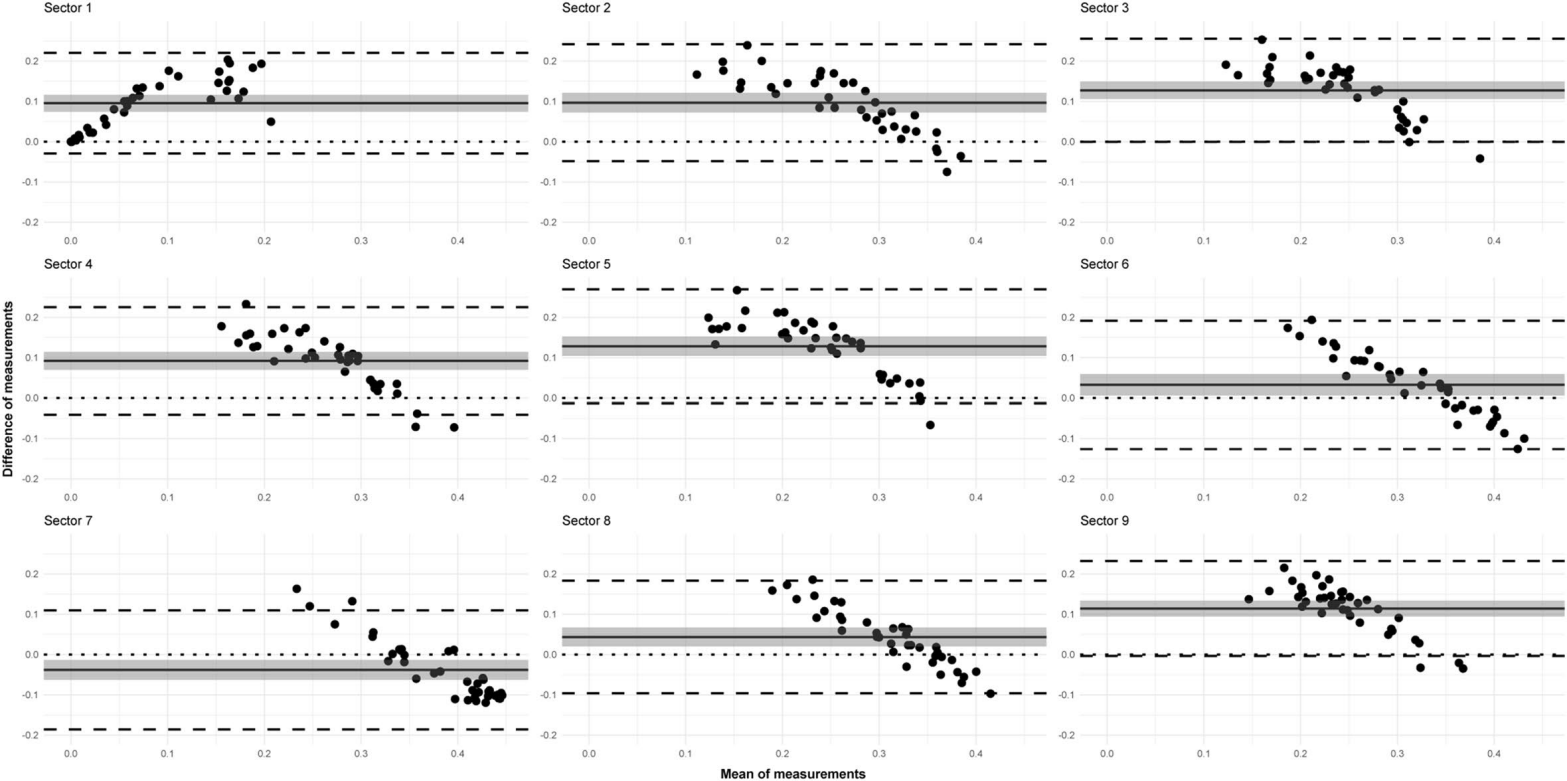
Mexican hat filter and Shanbhag thresholding comparison using the VD parameter. Bland–Altman plots in the 9 ETDRS sectors (see Fig. 3 for the explanation of the sectors). The x axis represents the mean of measurements, the y axis represents the difference of measurements for each graph, individually. The solid line indicates the mean of the differences; the shade around the solid line indicates the 95% confidence interval of mean difference; the upper and lower dashed lines indicate the upper and lower limits of agreement (LA), whereas the dotted line indicates zero.

Regarding the VD parameter, the differences were not so consistent (Fig. 6). The correlation between the means and differences of the two methods was negative in sectors 2, 3, 4, 5, and 9. In contrast, in sectors 6, 7 and 8, in the case of a low mean value, the values of MHF were higher than those of ST, while in the case of higher means, measurements of ST were also higher. The mean of differences was positive in all sectors except for sector 7 (in agreement with the boxplots). The lower limit of agreement contained zero in almost every sector; however, the mean of differences was significantly different from zero in all sectors based on the 95% confidence interval.

**Figure 7:**
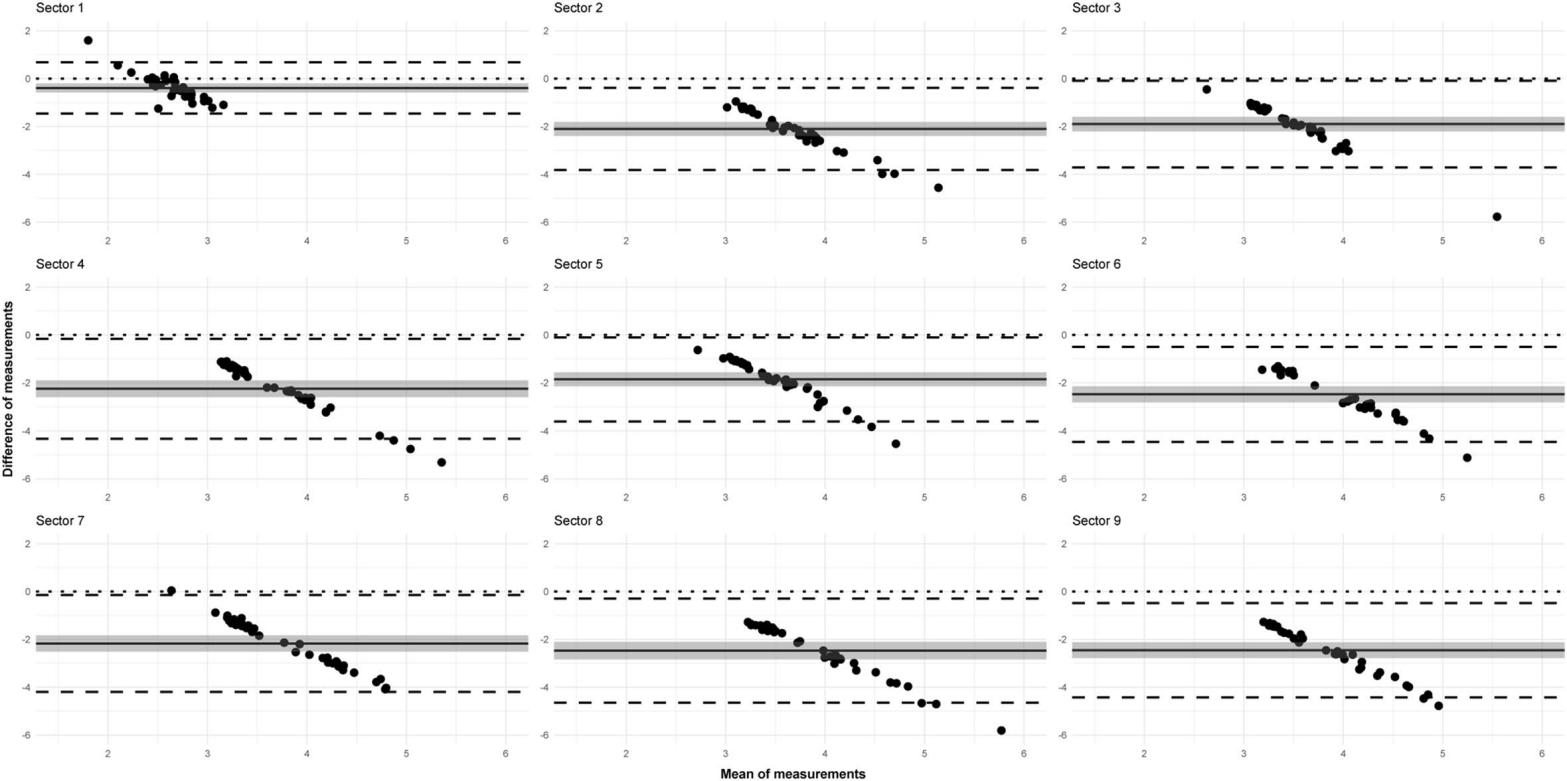
Mexican hat filter and Shanbhag thresholding comparison using the VDI parameter. Bland–Altman plots in the 9 ETDRS sectors (see Fig. 3 for the explanation of the sectors). The x axis represents the mean of measurements, the y axis represents the difference of measurements for each graph, individually. The solid line indicates the mean of the differences; the shade around the solid line indicates the 95% confidence interval of mean difference; the upper and lower dashed lines indicate the upper and lower limits of agreement, and the dotted line indicates zero.

In respect of the VDI parameter, in agreement with the previous descriptive results, values of ST were higher than those of MHF, and the differences were in the negative range (Fig. 7).

Based on this observation and the shape of the point cloud we could conclude that the correlation between the means and differences of the two methods is positive, meaning the higher the mean of measurements is, the larger the difference will be. The upper limit of agreement did not contain zero in any sector (except for sector 1). The mean of differences was significantly different from zero in all sectors based on the 95% confidence interval.

## Discussion

Since OCT angiography became part of the daily clinical practice, it is possible to analyze the macular vasculature and retinal vascular density non-invasively. OCT technology has developed very quickly in the past two decades, although there are still limitations regarding image processing and quantification of OCTA images. OCTA devices are different, they utilize different components of the OCT signal, use different segmentation algorithms, and also different post-processing methods. Retinal VAD analysis based on OCTA images came into focus in the past few years, and before the introduction of automated VAD analyzing programs, several studies discussed the advantages and disadvantages of different VAD quantification tools and OCTA devices^3–10^. There are additional software programs (such as MATLAB, ImageJ and Angiotool^5,21,22^) which enable semi-manual VAD measurements of binarized and/or skeletonized OCTA images, and they are often used in ophthalmological studies^3,23–25^. Corvi et al. compared 5 different threshold tools in ImageJ. Simultaneously, they have also recorded the threshold grey level value of each method and modified brightness compared to the default. They have found that the type of the thresholding method and changing the brightness and/or the contrast significantly impacts the outcomes of vascular density measurements^26^. Monares-Zepeda et al. compared the 3 × 3 and 6 × 6 mm scanning protocols of the same OCT device (Zeiss Cirrus Angioplex) to determine if their vascular density metrics could be interchanged. They concluded that different protocols measure different macular areas and therefore their metrics should not be interchanged for follow-up^27^. In another recent study, Lu et al. compared 4 different OCT devices in healthy eyes regarding their FAZ area, VAD, and fractal dimension (FD) measurements. They have found that FAZ measurements were consistent across all four devices, while VAD and FD measurements showed significant variability calculated by the different devices^28^. Chen et al. compared quantitative changes in macular parameters in patients with diabetes with two different OCTA machines^4^. In their research the two devices were largely inconsistent in terms of SkD and VD parameters. They used the size comparison index (SCI), to simplify the comparison of OCTA parameter values between the two OCTA devices^4^.

Prior to this current study, our research team explored several post-processing techniques using the ImageJ software in a preliminary pilot. Since Mexican hat filtering (MHF), was already applied in a handful of ophthalmological studies^6,29–34^, this technique was chosen as a basis for comparison.

During our exploration, we identified Shanbhag thresholding (ST) as promising candidate due to its potential for visualizing capillary drop-out in affected areas more effectively than Mexican hat filtering. To the best of our knowledge, ST has only been mentioned in one paper for the processing of OCTA images^35^ among several other thresholding techniques and has not received a lof of attention since.

Our study focused on comparing retinal vascular metrics of healthy subjects obtained using two post-processing methods on the same images captured on the same OCTA system. Our first method with MHF was based on a previous study published by Kim et al.^6^ They quantified changes in retinal microvasculature in different severity groups of patients with diabetic retinopathy (DR) by using spectral-domain optical coherence tomography angiography (SD-OCTA). Their results showed that binarized and skeletonized images both demonstrated areas of vascular abnormalities more clearly than the original SD-OCTA images^6^. MHF is a top-hat filter, it allows vascular density and diameter measurements, and it is a commonly used algorithm in ophthalmological studies assessing VAD^6,29–34^. ST was introduced by Shanbhag^12^ and according to our literature research, before the recent publication by Arrigo et al.^35^, the use of this technique for the purpose of medical image analysis was only published in two papers, first for assessing nerve echogenicity^36^ and second for dermatoscopy images^37^. Our research team chose this latter technique for the comparisons to examine the potential clinically relevant advantages that ST might have, as described in more details below.

There are some key differences between these two methods. MHF determines an average grey level threshold based on the whole image and classifies the brightness of the pixels in proportion to this threshold (fixed threshold). On the other hand, ST examines the surroundings of each pixel, and sets the brightness of a given pixel based on this information (dynamic threshold). As a consequence, MHF may show slightly more capillaries than otherwise visible on the original OCTA image, which is why it results in a more regular-looking vascular network. ST shows the bigger vessels sharper and thicker, and it makes more capillaries disappear, therefore we can see more black areas on the ST binarized images.

Although it would seem that MHF shows the anatomical vascular network more realistically, the noise filter function of both methods has almost the same efficiency, but it has the opposite effect: MHF shows more capillaries than the reality, while ST makes some capillaries disappear. The above detailed mathematical function of ST may make this method more capable to separate eyes with decreased VAD from healthy eyes, because the areas with decreased VAD have more contrast, than with MHF. However, this assumption is solely based on the mathematical definition of ST, and needs to be confirmed on a cohort of patients with retinal pathology.

We presume from the above that due to thresholding and noise filter properties, both methods may produce image artefacts during binarization: MHF may result in false flow, and ST may result in flow void^38^. After comparing the binarized images with the original image, we suspect that the extent of artefact production is not equal: false flow by MHF is considerably lower than flow void by ST. This finding is only based on our observation as the real VAD is not known.

While using MHF, the FAZ needs to be outlined manually, while ST makes it disappear automatically. We could see on the OCTA images, that the FAZ assigned by ST was considerably larger than the manually outlined FAZ with MHF. This difference originates from the different thresholding of the two methods. However, we examined young, healthy subjects with normal vascular networks and normal-sized FAZ; also, the images binarized by MHF appeared to be more similar to the original OCTA image than that by ST. It should be noted that our study did not focus on FAZ measurements; therefore, these statements are merely of qualitative nature.

Additionally, we compared the two techniques based on how fragmented the continuous vascular network of our healthy subjects will become after the application of the two filters. We presume that a less fragmented vasculature on binarized OCTA images is closer to real anatomy. With MHF, we observed more fragmented segments than with ST.

As mentioned earlier, our study included healthy participants only. Without the comparison of different patient cohorts, it is difficult to tell whether ST should be generally recommended over MHF or vice-versa; however, we assume that MHF could have merit when dealing with pathologies affecting the FAZ. This assumption needs to be confirmed in future studies including patient cohorts with retinal pathologies.

From the perspective of practical use, we found no differences. Both methods were easily applicable, training of the non-automated steps of both techniques to a non-trained co-author took a total of 1 h and the analysis of one data set took 4–5 min.

With the introduction of automated VAD analyzing software programs (such as Angioplex Metrix by Zeiss, AngioAnalytics by Optovue, Angioscan by Nidek), they are becoming frequently used tools in the clinical practice, but none of them measures VDI automatically. VDI includes information from both binarized and skeletonized images, and it has special importance in certain retinal disorders^6,39,40^. SkD also plays an important role in examining vessels with different diameters. Lei et al. examined the macular superficial capillaries and large retinal vessels separately. They have measured capillary density by subtracting the large vessels from total vasculature. After statistical processing, it was shown that large vessels had a tendency of enlargement while the superficial capillaries decreased dramatically by the increase of diabetic retinopathy (DR) severity. Vessel length density (VLD)—which is equal to SkD—showed the strongest correlation with the severity of DR. Since the cell injury effect of hyperglycemia initially impairs the pericytes and endothelial cells of capillaries, SkD could be a potential parameter in the evaluation of diabetic retinopathy^40^.

Based on the above studies, we believe that automated quantification programs should probably calculate all 3 VAD parameters in both layers during a detailed examination of retinal perfusion.

Due to the different algorithms and nomenclature used by semi-automated and automated programs, cross-comparisons are nearly impossible. Standardization of the algorithms and programs is unlikely in the near future due to the relatively large number of manufacturers and their use of different technologies. Unfortunately, there is no gold standard for VAD analysis, no consensus in the literature, and no unified nomenclature for the investigated parameters. Munk et al. works towards the development of a nomenclature consensus for OCTA findings in retinal vascular diseases. A consensus was reached regarding several areas and topics of VAD measurement, but they also discussed that nomenclatures describing quantification of OCTA-based measures are still confusing, and there is a need for uniform terminology among the software programs of different manufacturers^41^. It would also be essential to create a robust normative database for vascular density measurements of healthy subjects, but due to the differences detailed earlier, this seems to be a very demanding task. In a recent publication, Tan et al. reported a normative database for 12 × 12 mm OCTA images involving 138 healthy Asian subjects, divided into 4 age groups. They used a swept-source OCT system, segmented the large vessels with U-NET, a deep learning architecture, and segmented the capillaries by a moving window scheme^42^.

The present study has several limitations. First, it should be noted that all our study participants were white Caucasians; therefore, the possibility of ethnicity as a confounding factor is excluded. Second, we focused our analysis of the vascular density parameters on the two aforementioned semi-manual methods, and no comparisons were made with other quantification tools. Third, we included a relatively small number of healthy participants; therefore, our results should be confirmed on larger cohorts. In order to be able to establish clinically relevant differences, these cohorts should include patients with retinal pathology.

## Conclusions

In this study, we processed the same OCTA images with two different methods. Both Mexican hat filtering and Shanbhag thresholding allowed the investigation of VD, SkD, and VDI in the superficial vascular layer in all sectors of the macula, with numerous differences. Noise level reduction was almost identical, the Shanbhag method broke the vascular network into fewer pieces, and both methods showed different artefacts. Regarding the examined vascular parameters, the two methods produced largely inconsistent results, meaning that they cannot be used interchangeably. This is in line with previous similar studies, that concluded that different VAD assessment methods are not cross-comparable, and even a minor change in processing parameters can lead to different final results. Further studies are required to determine if these inconsistent results are clinically meaningful and which algorithm is superior as there is no gold standard for vascular density analysis and the actual density is not known. For longitudinal monitoring purposes, use of the same image processing method is recommended, and an expert consensus is required to determine the best processing algorithm. Our study provides a detailed exploration of the Shanbhag thresholding technique, which is not commonly employed in OCTA post-processing in ophthalmology, potentially laying the groundwork for future studies in this area.

## Data Availability

The datasets used and/or analyzed during the current study are available from the corresponding author on reasonable request.
